# miR-136 Regulates the Proliferation and Adipogenic Differentiation of Adipose-Derived Stromal Vascular Fractions by Targeting *HSD17B12*

**DOI:** 10.3390/ijms241914892

**Published:** 2023-10-04

**Authors:** Jianhua Liu, Yutong Che, Ke Cai, Bishi Zhao, Liying Qiao, Yangyang Pan, Kaijie Yang, Wenzhong Liu

**Affiliations:** 1 College of Animal Science, Shanxi Agricultural University, Jinzhong 030801, China; ljhbeth@sxau.edu.cn (J.L.); cyy126@163.com (Y.C.); 18574385119@163.com (K.C.); zhaobishi2021@163.com (B.Z.); liyingqiao@sxau.edu.cn (L.Q.); panyy@sxau.edu.cn (Y.P.); kjyang@sxau.edu.cn (K.Y.); 2 Key Laboratory of Farm Animal Genetic Resources Exploration and Breeding of Shanxi Province, Jinzhong 030801, China

**Keywords:** sheep, miR-136, HSD17B12, SVFs, proliferation, differentiation

## Abstract

Fat deposition involves the continuous differentiation of adipocytes and lipid accumulation. Studies have shown that microRNA miR-136 and 17β-hydroxysteroid dehydrogenase type 12 (*HSD17B12*) play important roles in lipid accumulation. However, the regulatory mechanism through which miR-136 targets *HSD17B12* during ovine adipogenesis remains unclear. This study aimed to elucidate the role of miR-136 and *HSD17B12* in adipogenesis and their relationship in ovine adipose-derived stromal vascular fractions (SVFs). The target relationship between miR-136 and *HSD17B12* was predicted and confirmed using bioinformatics and a dual-luciferase reporter assay. The results showed that miR-136 promoted proliferation and inhibited adipogenic differentiation of ovine SVFs. We also found that *HSD17B12* inhibited proliferation and promoted adipogenic differentiation of ovine SVFs. Collectively, our results indicate that miR-136 facilitates proliferation and attenuates adipogenic differentiation of ovine SVFs by targeting *HSD17B12*. These findings provide a theoretical foundation for further elucidation of the regulatory mechanisms of lipid deposition in sheep.

## 1. Introduction

Excess lipids are stored in the animal body as adipose tissue. In sheep, excess fat is deposited in the tail, providing energy during adverse conditions. However, in modern farming, excessive fat deposits in the tail do not provide economic benefits [1]. Therefore, exploring the regulatory mechanisms underlying fat deposition in sheep is necessary.

Fat deposition is regulated by several factors, including circular RNAs, microRNAs (miRNAs), and long non-coding RNA. miRNAs are endogenous small non-coding RNAs (20–24 nucleotides) that bind to target gene mRNAs, promote their degradation, inhibit their translation at the transcriptional level, and affect various cellular properties and physiological processes in vivo [2,3,4]. Numerous recent studies have shown that several miRNAs play important roles in adipogenesis. miR-127 targets mitogen-activated protein kinase 4 to promote porcine adipocyte proliferation [5]. miR-130b inhibits the lipid accumulation of porcine preadipocytes by directly targeting the peroxisome proliferator-activated receptor gamma (PPARγ) [6]. In sheep, miR-128-1-5p promotes the expression of lipogenic marker genes and the formation of lipid droplets by targeting the Kruppel-like transcription factor 11 5′-UTR [7]. miR-301a inhibits adipogenic differentiation of ovine preadipocytes by targeting homeobox C8 [8]. miR-136 expression is lower in large white pigs with higher back fat deposition [9]. Furthermore, the expression of miR-136 in subcutaneous adipose tissue was significantly higher than in sheep perirenal adipose tissue [10]. However, the regulatory mechanism by which miR-136 targets *HSD17B12* during ovine adipogenesis remains unclear. Based on previous studies, we speculated that miR-136 might play a role in the proliferation and adipogenic differentiation of ovine stromal vascular fractions (SVFs).

Based on the mechanism of action of miRNAs, we made bioinformatics-based predictions and found that *HSD17B12* was the target gene of miR-136. *HSD17B12* is a member of the 17β-hydroxysteroid dehydrogenases, a class of enzymes that catalyze the interconversion of active and inactive steroid hormones [11]. *HSD17B12* is widely expressed in animal kidneys, livers, and ovaries [12]. In addition, *HSD17B12* has many important biological functions, including fatty acid metabolism, sex hormone production, and cell cycle regulation [12,13]. Notably, the expression of *HSD17B12* did not increase in the livers of transgenic mice overexpressing sterol regulatory element-binding proteins (SREBP) [14]. However, another study found that the expression of *HSD17B12* and other SREBP-regulated genes, such as fatty acid synthase, significantly increased in HepG2 cells where *SREBP-1* was activated [15]. However, the results of these two studies were inconsistent. These inconsistencies may be due to species differences; further research is required to confirm this hypothesis. Moreover, interference with *HSD17B12* expression inhibits the proliferation of breast cancer cells [16]. In human adipocytes, *HSD17B12* downregulates the lipoprotein lipase expression and affects adipocyte maturation and lipid accumulation [17]. However, there is a dearth of studies on the precise function of *HSD17B12* in ovine adipogenesis.

In this study, we investigated the target relationship between miR-136 and *HSD17B12*. We also explored the effects of miR-136 and *HSD17B12* on the proliferation and adipogenic differentiation of ovine SVFs and their possible mechanism of action. This study aimed to elucidate the mechanism of action of miR-136 in the proliferation and adipogenic differentiation of ovine SVFs, thereby providing a foundation for studying adipogenesis regulation by miRNAs.

## 2. Results

### 2.1. Identification of Ovine SVFs

**Figure 1:**
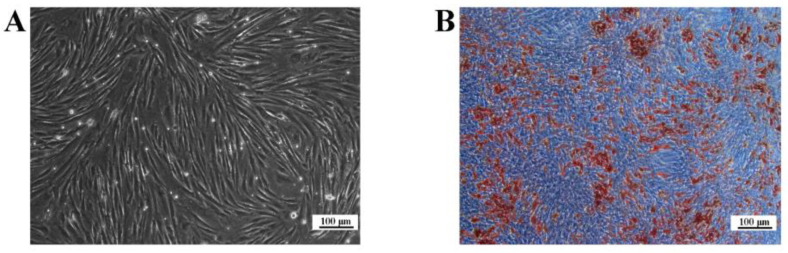
Ovine SVFs cultured in vitro. (**A**) Ovine primary SVFs were cultured for 6 days (**B**) ORO-stained SVFs after the induction of differentiation for 10 days. Blue indicates the background, while brown indicates lipid droplets. Scale bars: 100 μm.

Isolated and cultured ovine SVFs are spindle-shaped (Figure 1A). Oil Red O (ORO) staining showed that more lipid droplets were produced after 10 days of differentiation induction (Figure 1B). In conclusion, these cells were successfully isolated and used in subsequent experiments.

### 2.2. miR-136 Targets HSD17B12 3′-UTR

**Figure 2:**
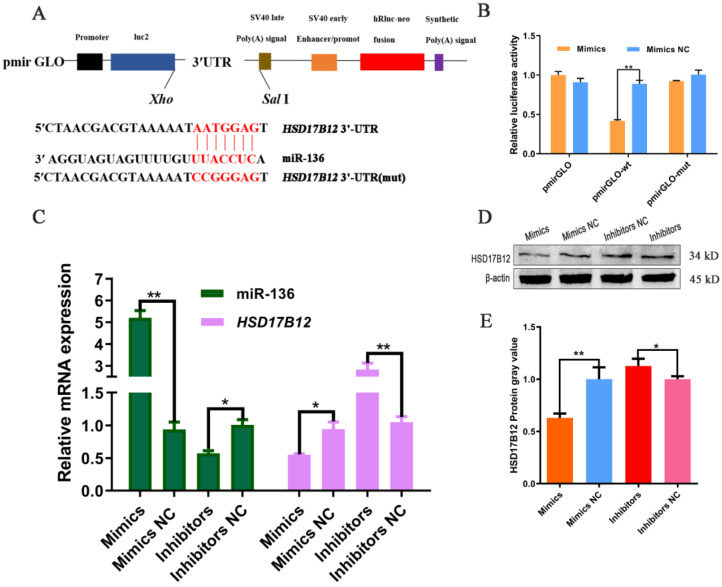
miR-136 binds the 3′-UTR of *HSD17B12* mRNA. (**A**) Schematic illustration of the *HSD17B12* 3′-UTR-wt and *HSD17B12* 3′-UTR-mut luciferase vectors. (**B**) The relative luciferase activities were determined in 293 T cells after transfection with a black vector, *HSD17B12* 3′-UTR-wt or *HSD17B12* 3′-UTR-mut and miR152 mimic or NC mimic, respectively. (**C**) The *HSD17B12* mRNA expression in ovine SVFs that overexpress or inhibit miR-136. (**D**,**E**) *HSD17B12* protein expression in ovine SVFs that overexpress or inhibit miR-136. * *p* < 0.05 and ** *p* < 0.01.

Based on bioinformatics analysis (Figure S1), we predicted that a binding site for miR-136 is present on *HSD17B12* 3′-UTR (Figure 2A). Dual-luciferase reporter assays confirmed this hypothesis (Figure 2B). miR-136 significantly reduced the luciferase activity of reporters containing the *HSD17B12* 3′-UTR (*p* < 0.05), whereas no significant difference was observed between the mutant and blank vectors. Furthermore, qPCR results (Figure 2C) showed that after the overexpression of miR-136, the *HSD17B12* mRNA expression was significantly downregulated (*p* < 0.05). After interference with miR-136, the *HSD17B12* mRNA expression significantly increased (*p* < 0.01). Western blotting results (Figure 2D,E) showed that the HSD17B12 protein expression significantly decreased (*p* < 0.01) after miR-136 overexpression. After interfering with miR-136, the HSD17B12 protein expression was significantly upregulated (*p* < 0.05). These results indicate that the miR-136 seed region can bind to the 3′-UTR of *HSD17B12* and that miR-136 negatively regulates *HSD17B12* expression.

### 2.3. miR-136 Promotes the Proliferation of Ovine SVFs

**Figure 3:**
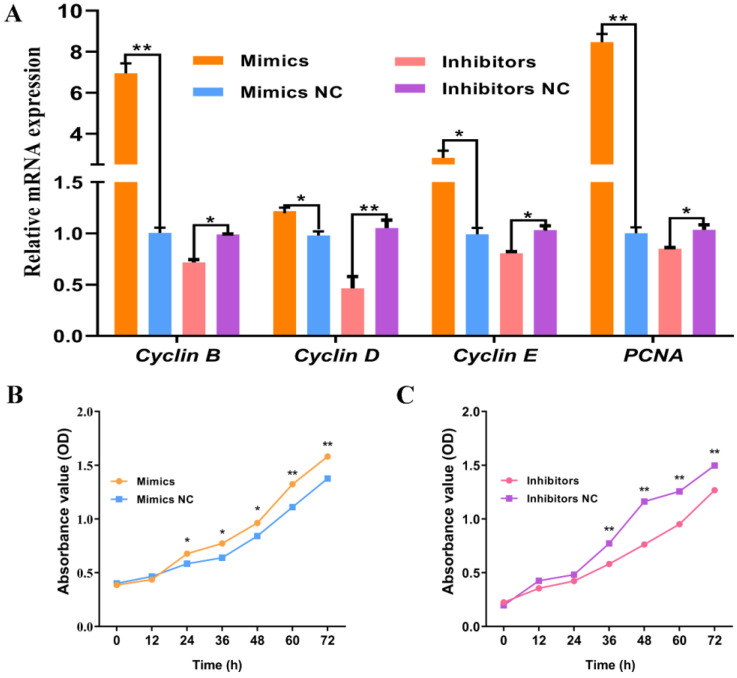
Effect of miR-136 on ovine SVF proliferation. (**A**) The mRNA expression of four proliferation marker genes in ovine SVFs that overexpress or inhibit miR-136. (**B**,**C**) Cell proliferation analysis in ovine SVFs overexpressing or inhibiting miR-136. * *p* < 0.05 and ** *p* < 0.01.

To understand the function of miR-136 on the proliferation of ovine SVFs, SVFs transfected with miR-136 mimics or miR-136 inhibitors were collected two days later. The mRNA expression of proliferation markers *cyclin B*, *cyclin D*, *cyclin E*, and *PCNA* was determined using qPCR (Figure 3A). The results showed that the mRNA expression of *cyclin D* and *cyclin E* was significantly upregulated compared to the transfected miR-136 mimic NC (*p* < 0.05), and the mRNA expression of *cyclin B* and *PCNA* was significantly upregulated (*p* < 0.01). In contrast, after transfection with miR-136, the mRNA expression of *cyclin B*, *cyclin E*, and *PCNA* was significantly downregulated (*p* < 0.05), and *cyclin D* mRNA expression was significantly downregulated (*p* < 0.01). A cell counting kit 8 (CCK-8) was used to determine the activity of cells at different proliferation stages (Figure 3B,C). The results showed that ovine SVFs transfected with miR-136 mimics proliferated for 24 h, and the cell proliferation rate was significantly higher than that of cells transfected with miR-136 mimic NC (*p* < 0.05). The degree of increase reached a significant level at 60 h (*p* < 0.01). After transfection with miR-136 inhibitors, the proliferation rate of the cells decreased and reached a significant level after 36 h (*p* < 0.01). These results suggested that miR-136 enhanced the proliferation of ovine SVFs.

### 2.4. miR-136 Inhibits the Adipogenic Differentiation of Ovine SVFs

**Figure 4:**
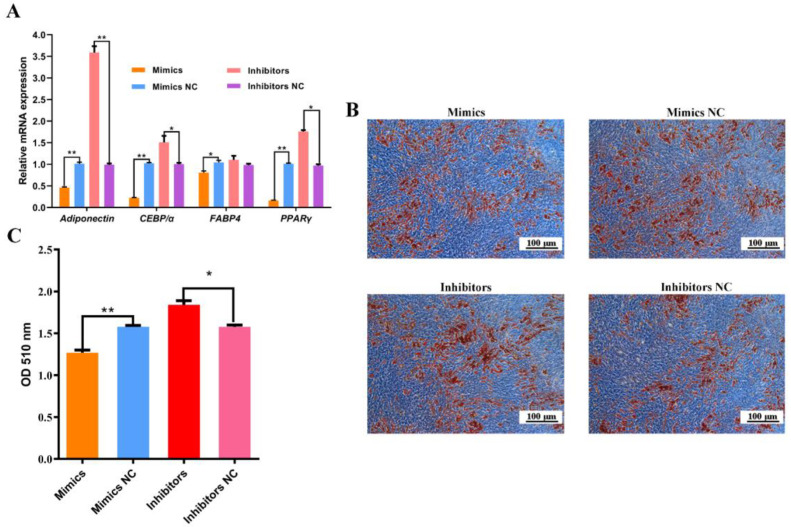
Effect of miR-136 on adipogenic differentiation of ovine SVFs. (**A**) mRNA expression of four lipogenic genes in ovine SVFs that overexpress or inhibit miR-136. (**B**) Ovine SVFs were stained with ORO on day 10 after the induction of differentiation. (**C**) Triacylglycerol concentration in ovine SVFs that overexpress or inhibit miR-136. * *p* < 0.05 and ** *p* < 0.01. Blue indicates the background, while brown indicates lipid droplets. Scale bars: 100 μm.

We investigated the effect of miR-136 on the adipogenic differentiation of ovine SVFs. The *FABP4* mRNA expression significantly decreased (*p* < 0.05), and the mRNA expression of *PPARγ*, *C/EBPα,* and *adiponectin* significantly decreased (*p* < 0.01) after the transfection of miR-136 mimics (Figure 4A). In contrast, the mRNA expression of *C/EBPα* and *PPARγ* were significantly upregulated (*p* < 0.05), and the *adiponectin* mRNA expression was significantly upregulated (*p* < 0.01) after the transfection of miR-136 inhibitors. However, the *FABP4* mRNA expression was not significant between groups. Cells transfected with miR-136 mimics accumulated fewer lipid droplets, whereas those transfected with miR-136 inhibitors accumulated more (Figure 4B). The triglyceride determination (Figure 4C) showed that the triglyceride content significantly decreased after transfection with the miR-136 mimic (*p* < 0.01) and significantly increased after transfection with the miR-136 inhibitor (*p* < 0.05). These results implied that miR-136 inhibited the adipogenic differentiation of ovine SVFs.

### 2.5. HSD17B12 Suppresses the Proliferation of Ovine SVFs

**Figure 5:**
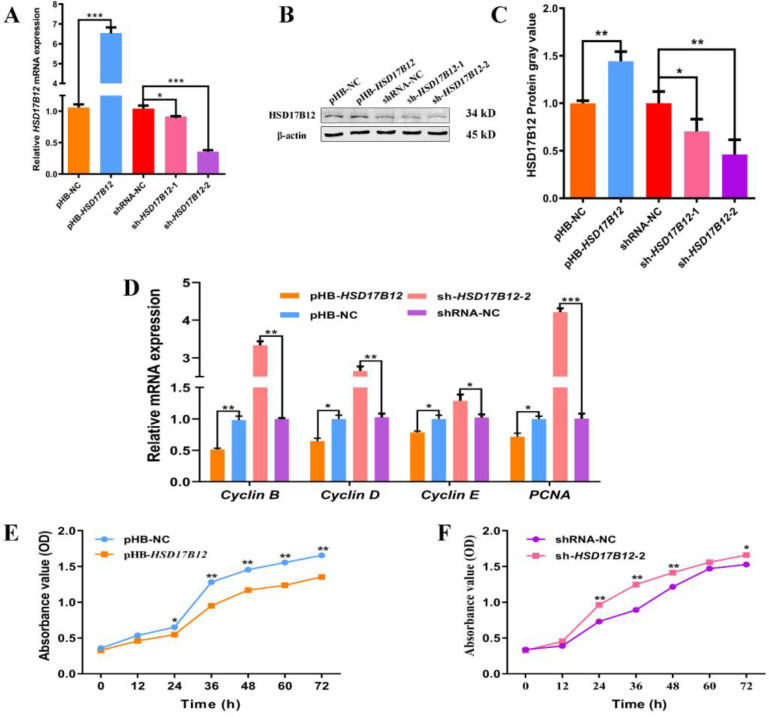
Effect of *HSD17B12* on ovine SVF proliferation. (**A**) mRNA expression of *HSD17B12* in ovine SVFs that overexpress or interfere with *HSD17B12*. (**B**,**C**) HSD17B12 protein expression in ovine SVFs that overexpress or interfere with *HSD17B12*. (**D**) mRNA expression of four proliferation marker genes in ovine SVFs that overexpress or interfere with *HSD17B12*. (**E**,**F**) Cell proliferation analysis in ovine SVFs that overexpress or interfere with *HSD17B12*. * *p* < 0.05, ** *p* < 0.01, and *** *p* < 0.001.

To investigate the effect of *HSD17B12* on the proliferation of ovine SVFs, we either overexpressed or disrupted *HSD17B12* expression in ovine SVFs by packaging lentiviruses. Overexpressing and interfering with *HSD17B12* significantly increased and decreased its expression in ovine SVF cells at both the mRNA (Figure 5A) and protein (Figure 5B,C) levels. Moreover, sh-*HSD17B12*-2 was more effective than sh-*HSD17B12*-1. Therefore, sh-*HSD17B12*-2 was used for subsequent experiments. Cells cultured for two days were collected, and the mRNA expression of proliferation markers *cyclin B*, *cyclin D*, *cyclin E*, and *PCNA* was determined using qPCR (Figure 5D). The results showed that overexpression of *HSD17B12* highly downregulated the *cyclin* mRNA expression (*p* < 0.01), whereas the mRNA expression levels of *PCNA*, *cyclin D*, and *cyclin E* were significantly downregulated (*p* < 0.05). In contrast, the expression of *cyclin B* (*p* < 0.01), *cyclin D* (*p* < 0.01), and *PCNA* (*p* < 0.001) mRNA was significantly upregulated, and the *cyclin* mRNA expression was significantly upregulated (*p* < 0.05) after the knockdown of *HSD17B12*. The CCK-8 results showed that the proliferation rate of ovine SVFs overexpressing *HSD17B12* was significantly lower than that of the pHB-NC group (*p* < 0.05) after 24 h of proliferation (Figure 5E), and this reduction reached a significant level (*p* < 0.01) at the subsequent four time points. After the inhibition of *HSD17B12* (Figure 5F), the proliferation rate of cells was higher than that of the shRNA-NC group and reached a highly significant level (*p* < 0.01) at 24 h. These results indicated that *HSD17B12* suppressed the proliferation of ovine SVFs.

### 2.6. HSD17B12 Facilitates the Adipogenic Differentiation of Ovine SVFs

**Figure 6:**
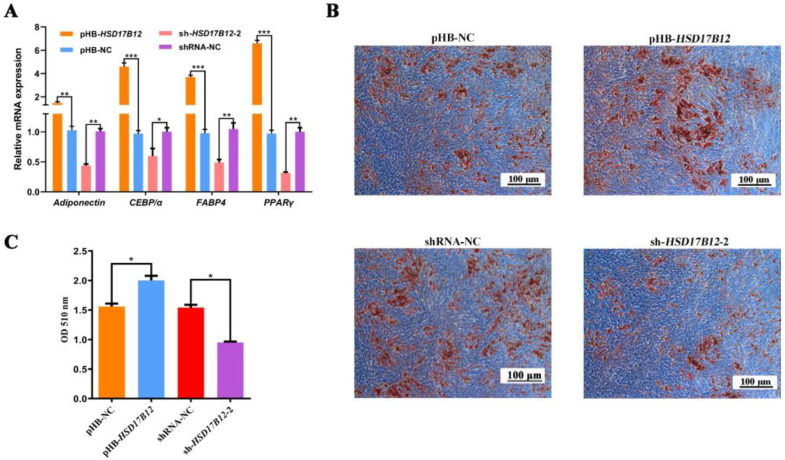
Effect of HSD17B12 on adipogenic differentiation of ovine SVFs. (**A**) mRNA expression of four lipogenic genes in ovine SVFs that overexpress or interfere with HSD17B12. (**B**) Ovine SVFs were stained with ORO on day 10 after the induction of differentiation. (**C**) Triacylglycerol concentration in ovine SVFs that overexpress or interfere with HSD17B12. * *p* < 0.05, ** *p* < 0.01, and *** *p* < 0.001. Blue indicates the background, while brown indicates lipid droplets. Scale bars: 100 μm.

We also explored the function of *HSD17B12* during the adipogenic differentiation of ovine SVFs. After *HSD17B12* overexpression, the *PPARγ* mRNA expression was significantly upregulated (*p* < 0.01), and the mRNA expression of *C/EBPα*, *adiponectin*, and *FABP4* was significantly upregulated (*p* < 0.001) (Figure 6A). After the knockdown of *HSD17B12*, the mRNA expression of *PPARγ*, *FABP4*, and *adiponectin* was significantly downregulated (*p* < 0.01), and the *C/EBPα* mRNA expression was remarkably downregulated (*p* < 0.05). The ORO staining results (Figure 6B) showed that the accumulation of lipid droplets in the pHB-*HSD17B12* group was significantly higher than that in the pHB-NC group and that the shRNA-*HSD17B12*-2 group accumulated fewer lipid droplets than the shRNA-NC group. In addition, the results of the triglyceride determination (Figure 6C) showed that the triglyceride content significantly increased after *HSD17B12* overexpression (*p* < 0.05), and the triglyceride content significantly decreased after interference with *HSD17B12* (*p* < 0.05). Collectively, these results demonstrate that *HSD17B12* promotes adipogenic differentiation and lipid accumulation in ovine SVFs.

## 3. Discussion

Adipose tissue includes adipocytes and other types of cells called SVF cells [18]. In a previous study, we isolated SVF cells from ovine back adipose tissue through collagenase digestion, and the cultured SVFs were spindle-shaped or triangular [19], which is consistent with our isolated SVFs. A large number of lipid droplets were observed in adipogenic-induced differentiated SVFs stained with ORO [19]. We used this method to identify the adipogenic differentiation ability of ovine SVFs, and the results showed that isolated SVFs produce large amounts of lipid droplets. Therefore, the SVFs isolated in this study can be used to study molecular functions associated with adipogenesis in vitro.

miRNAs play important roles in adipogenesis. Thus, miR-136 is a potential regulator of adipogenesis [10]. miRNAs can bind to the mRNAs of their target functional genes in a partially or fully complementary manner and promote their degradation or inhibit their translation at the post-transcriptional level [2]. Indeed, our study revealed that miR-136 and *HSD17B12* have a targeting relationship and that miR-136 negatively regulates *HSD17B12* mRNA and protein expression. Therefore, we speculated that miR-136 likely affects adipogenesis by regulating *HSD17B12* expression in sheep.

Adipogenesis involves two important biological processes: the proliferation and differentiation of adipocytes [20]. Many studies have demonstrated that the same miRNAs have opposing effects on adipocyte proliferation and differentiation. In other words, if a miRNA plays an inhibitory role in cell proliferation, it promotes cell differentiation. For example, miR-146b inhibits the proliferation and promotes the differentiation of porcine intramuscular preadipocytes [21], whereas miR-125a-5p promotes the proliferation of 3T3-L1 adipocytes and inhibits their differentiation [22]. miRNAs have also been reported to play the same roles in adipocyte proliferation and differentiation. For example, miR-146a-5p targets SMAD family member 4 and tumor necrosis factor receptor-related factor 6, inhibiting the proliferation and differentiation of porcine intramuscular preadipocytes [23]. In the present study, miR-136 promoted SVF proliferation and inhibited adipogenic differentiation and lipid accumulation. However, whether miR-136 modulates the proliferation and adipogenic differentiation of ovine SVFs by regulating the expression of *HSD17B12* remains unclear.

HSD17B12 is a multifunctional enzyme highly expressed in the brown and white adipose tissues of mice, and upregulation of *HSD17B12* induces fatty acid elongation [24]. Additionally, interfering *HSD17B12* inhibited the growth of breast cancer cells, whereas supplementation with arachidonic acid completely restored growth [25]. These studies suggest that *HSD17B12* is directly or indirectly involved in fat metabolism. Our study showed that *HSD17B12* inhibited proliferation and promoted adipogenic differentiation of ovine SVFs. Consistent with this, the overexpression of *HSD17B12* in bovine mammary epithelial cells inhibits cell proliferation and induces apoptosis [26]. Considering the target relationship between *HSD17B12* and miR-136 mentioned above, we believe that the influence of *HSD17B12* on ovine SVF development is regulated by miR-136. Genes are regulated by multiple miRNAs. In bovine mammary epithelial cells, the *HSD17B12* mRNA and protein expression significantly decreased because of the overexpression of miR-152 [26]. miRNAs can also target multiple genes [27]. For example, miR-136-3p inhibits the occurrence of gliomas by targeting *KLF7* in vivo [28]. The other target genes of miR-136 or miRNAs regulating *HSD17B12* need to be supplemented through further studies. In addition, the functional study of miR-136 and *HSD17B12* in vivo is also a problem that we need to solve next.

In summary, miR-136 inhibits *HSD17B12* expression by binding to its 3′-UTR, thereby promoting proliferation and negatively regulating adipogenic differentiation of ovine SVFs. We elucidated the negative regulatory effect of *HSD17B12* on proliferation and the positive regulatory effect on adipogenic differentiation in ovine SVFs. This study provides a scientific basis for further understanding the regulatory mechanism of miRNAs in the fat metabolism of sheep.

## 4. Materials and Methods

### 4.1. Ethics Statement

All animal procedures were approved by the Animal Care and Ethics Committee of Shanxi Agricultural University, China (No. SXAU-EAW-2022S.UV.010009).

### 4.2. Isolation and Culture of Ovine SVFs

Healthy 3-month-old Guangling large-tailed sheep were sacrificed, and their tails were sterilized with 75% ethanol. Then, a small sterile piece of tail fat tissue was rinsed in 75% alcohol several times and placed in phosphate-buffered saline (PBS; Solarbio, Beijing, China) containing 1% penicillin–streptomycin. After cutting and digesting the tail fat tissue with 2 mg/mL collagenase type II (Solarbio, Beijing, China), the suspension filtered through 75 and 37.5 µm nylon meshes was inoculated onto a culture dish. The solution was shaken gently to distribute the cells evenly. Lastly, the culture dishes were placed in an incubator. After culturing for 6 h, we observed whether the cells adhered to the culture dish and replaced the medium with fresh 89% low-glucose Dulbecco’s modified Eagle’s medium (Biological Industries, Kibbutz Beit Haemek, Israel) containing 10% fetal bovine serum (Biological Industries, Kibbutz Beit Haemek, Israel) and 1% penicillin–streptomycin, and cultured for an additional 48 h. These are the cultured SVFs.

### 4.3. Adipogenic Induction and ORO Staining

If the SVFs were evenly distributed in the culture dish and reached approximately 85% confluence, the growth medium was replaced with an induction medium, which contains 10 mM rosiglitazone (Cayman Chemical, Ann Arbor, MI, USA), 1.4 mg/mL 3-isobutyl-1-methylxanthine (Solarbio, Beijing, China), 1 mg/mL dexamethasone (Solarbio, Beijing, China)), and 3 mg/mL bovine insulin (Solarbio, Beijing, China). This induction was maintained for 10 days of differentiation. The SVFs’ growth state and the generation of lipid droplets were continuously monitored.

When a large number of lipid droplets appeared in the cells, the induction of differentiation of ovine SVFs was stopped. ORO staining was used to identify the lipid droplet distribution. To stain the lipids, cells were washed with cold PBS three times and fixed in 4% paraformaldehyde overnight at 4 °C. The cells were then incubated with the ORO working solution for 30 min. After repeated cleaning with double-distilled water, images were obtained with a microscope (Leica, Wetzlar, Germany).

### 4.4. Target Gene Prediction and Luciferase Reporter Assays

The binding between miR-136 and *HSD17B12* was predicted using the online tools TargetScan, miRDB, and miRBase. AnnHyb 4.946 software was used to design two specific amplification primers for sheep *HSD17B12* 3′-UTR, insert appropriate restriction sites at both ends of the primers, and synthesize them (Thermo Fisher Scientific, Waltham, MA, USA). Tissue RNA was extracted and reverse-transcribed to obtain cDNA, which was used as a template for PCR amplification of the sheep *HSD17B12* 3′-UTR. The pmirGLO vector was digested with the restriction enzymes *Xho* I and *Sal* I. The cloned and purified target fragments were ligated with a pmirGLO linear vector to construct *HSD17B12* 3′-UTR wild (*HSD17B12* 3′-UTR-wt) and *HSD17B12* 3′-UTR mutant vectors (*HSD17B12* 3′-UTR-mut) using the ClonExpress Ultra One Step Cloning kit (C115-02, Vazyme, Jiangsu, China).

When the 293T cell density reached 70%, and the distribution was uniform, the transfection reagents, recombinant plasmids, miR-136 mimics, and miR-136 mimic NC were co-transfected. Luciferase activity was detected at 48 h using a Dual-Luciferase Reporter Assay System kit (Promega, Shanghai, China).

### 4.5. Transfection of Mir-136 Mimics and Inhibitors into Ovine Preadipocytes

Ovine SVFs were evenly seeded into 6-well plates. When the growth density reached approximately 75%, lipofectamine 3000 (Thermo Fisher Scientific, Waltham, MA, USA) was used to transfect the miR-136 mimic, miR-136 mimic NC, miR-136 inhibitors, and miR-136 inhibitor NC. After 6 h of transfection, the medium was replaced with a fresh one to continue the culture. After 48 h of culture, cells were collected for subsequent experiments.

### 4.6. Lentiviral Infection

Based on the predicted sequence of sheep *HSD17B12* mRNA (XM_004016421.4) published in GenBank, specific primers for the CDS region of HSD17B12 were designed using AnnHyb 4.946 software (Informer Technologies) (Table 1).

Primer sequences were synthesized by Thermo Fisher Scientific. The CDS of the ovine *HSD17B12* gene was cloned using sheep cDNA as a template. The pHBLV-CMVIE-ZsGreen-T2A-puro vector was digested to form a linear fragment, which was used to construct a recombinant plasmid. According to the seamless cloning kit instructions, the recovered and purified *HSD17B12* target fragment was ligated into a successfully digested pHBLV-CMVIE-ZsGreen-T2A-puro linear vector. Using BLOCK-iT RNAi Designer online software, two pairs of shRNA interference sequences of *HSD17B12* were designed and sent to Thermo Fisher for synthesis. The primer sequences are shown in Table 2. The annealed *HSD17B12*-shRNA was ligated into a linearized pHBLV-U6-ZsGreen-Puro vector.

The 293T cells were resuspended in a culture dish. The recombinant plasmid and two packaged plasmids, PMD2.g and psPAX2 (purchased from Hanbio, Shanghai, China), were transferred to 293T cells whose density reached 70%. The cell culture medium was collected 48 and 72 h after transfection and filtered through a disposable filter with a pore size of 0.45 μm. The virus solution prepared was collected and stored at 4 °C for later use. Ovine SVFs were cultured in six-well plates, and growth was observed to ensure that the cells in each well adhered evenly. The virus solution prepared was added when the cell density reached 60%. After 48 h of infection, the complete culture medium was replaced. The cells were observed for green fluorescence. The presence of green fluorescence indicated that the lentiviral infection was successful.

### 4.7. Quantitative Real-Time PCR (qRT-PCR)

Total RNA was extracted from cells using RNAiso Plus (Takara, Kusatsu, Japan). The M5 miRNA qPCR Assay kit (MF307-01; Mei5bio, Beijing, China) was used to determine the expression of miR-136 after transfection. The TB Green Premix Ex Taq II Master Mix (Takara, Kusatsu, Japan) was used to determine the expression of proliferation and differentiation marker genes. The expression of *β-actin* was used as an internal reference for coding genes, and *U6* was used as an internal reference to evaluate miR-301a levels. All primer sequences used for qRT-PCR analysis are listed in Table 3.

### 4.8. Cell Count Determination

When the ovine SVFs reached a density of 50%, cells were transfected with miR-136. Cells were harvested after treatment at seven time points from 0 to 72 h (0, 12, 24, 36, 48, 60, and 72 h). The CCK-8 solution and complete medium were diluted at a ratio of 1:10, added to the cells, and incubated for 2.5 h. The absorbance of the cells was measured at 450 nm in the dark.

### 4.9. Western Blotting

Total proteins were collected from the cell samples after lysis with a lysis buffer (RIPA buffer, Solarbio, Beijing, China) supplemented with protease inhibitors (Solarbio, Beijing, China), phosphatase inhibitors (Solarbio, Beijing, China), and phenylmethylsulfonyl fluoride (Solarbio, Beijing, China). Sodium dodecyl sulfate–polyacrylamide gel electrophoresis was performed on a 10% gel. After filtration through a nitrocellulose membrane (Solarbio), the membrane was sealed with 5% skim milk and treated with primary antibodies (anti-HSD17B12, 1:500, Thermo Fisher Scientific, Shanghai, China; anti-β-actin, 1:3000, Immunoway, Beijing, China) and secondary antibodies. Finally, the membranes were imaged using an Odyssey Clx Imaging System (LICOR, Lincoln, NE, USA) and quantified using Image-Pro Plus software (Media Cybernetics, Rockville, MD, USA).

### 4.10. Statistical Analyses

At least three biological replicates and three technical replicates were established for each group of experiments, and the experimental results were analyzed and plotted using GraphPad Prism 8.0 (GraphPad, San Diego, CA, USA). The qPCR results were calculated according to the 2^−^^ΔΔ*C*t^ algorithm, and the *t*-test and one-way analysis of variance were used to determine the significance. *p* < 0.05 was considered significant, and *p* < 0.01 was considered highly significant.

## Figures and Tables

**Table 1:** Cloning primers for *HSD17B12* CDS in sheep.

Genes	Sequences (5′→3′)	Sequences Length (bp)
*HSD17B12*-F	GATCTATTTCCGGTGAATTCTGAGGCCTGGTTGAAAGCCAT	939
*HSD17B12*-R	ACTAGTATCGATGGATCCTCGCTTAGCTGGCGCATCT	

Underlined parts are restriction sites.

**Table 2:** *HSD17B12* interference sequence primers.

Names	Sequences (5′→3′)
shRNA-1 F	GATCCGGACAAACTGAACCAGGTTTCTTCAAGAGAGAAACCTGGTTCAGTTTGTCCTTTTTTG
shRNA-1 R	AATTCAAAAAAGGACAAACTGAACCAGGTTTCTCTCTTGAAGAAACCTGGTTCAGTTTGTCCG
shRNA-2 F	GATCCGGACCAATGGATACTTCATCCTTCAAGAGAGGATGAAGTATCCATTGGTCCTTTTTTG
shRNA-2 R	AATTCAAAAAAGGACCAATGGATACTTCATCCTCTCTTGAAGGATGAAGTATCCATTGGTCCG

**Table 3:** qPCR primer names and sequences of marker genes.

Genes	Sequences (5′→3′)	Sequences Length (bp)
*Cyclin B*	F: CGATACTCCGTCTCCAAGCC	261
	R: AGCCAGTCAATCAGGATGGC	
*Cyclin D*	F: GATGCCAACCTCCTCAACGA	221
	R: GGAAGCGGTCCAGGTAGTTC	
*PCNA*	F: ATCAGCTCAAGTGGCGTGAA	231
	R: TGCCAAGGTGTCCGCATTAT	
*PPARγ*	F: ATCTTGACGGGAAAGACGAC	156
	R: AAACTGACACCCCTGGAAGAT	
*C/EBPα*	F: TCCGTGGACAAGAACAGCAA	137
	R: TCATTGTCACTGGTCAGCTCC	
*Adiponectin*	F: ATCCCCGGGCTGTACTACTT	129
	R: CTGGTCCACGTTCTGGTTCT	
*FABP4*	F: AAACTGGGATGGGAAATCAACC	109
	R: TGCTCTCTCGTAAACTCTGGTAGC	
*β-Actin*	F: TGATGATATTGCTGCGCTCG	194

## Data Availability

All data generated or analyzed during this study are included in this published article. The data that support the findings of this study are available from the corresponding author upon request.

## Supplementary Materials

The following supporting information can be downloaded at https://www.mdpi.com/article/10.3390/ijms241914892/s1.
