# Supplementary figures and images for: miR-136 Regulates the Proliferation and Adipogenic Differentiation of Adipose-Derived Stromal Vascular Fractions by Targeting HSD17B12
Source: Int J Mol Sci. 2023 Oct 4;24(19):14892. doi: 10.3390/ijms241914892 (PMC10573499; doi:10.3390/ijms241914892)

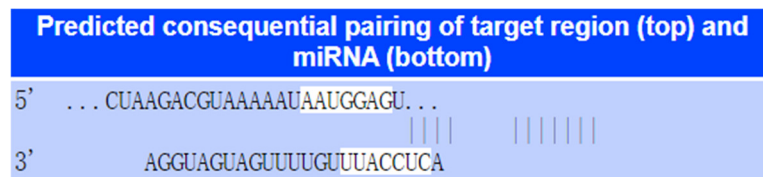

**Figure S1.** Target scan software predicts the binding site of miR-136 and *HSD17B12*.

Supplement: Supplementary file 1 [file ijms-24-14892-s001.zip › ijms-2597270-supplementary.pdf]
